# Supplementary material for: PAXX and its paralogs synergistically direct DNA polymerase λ activity in DNA repair
Source: Nat Commun. 2018 Sep 24;9:3877. doi: 10.1038/s41467-018-06127-y (PMC6155126; doi:10.1038/s41467-018-06127-y)
Supplement: Supplementary file 1 — Supplementary Information [file 41467_2018_6127_MOESM1_ESM.pdf]

**<u>SUPPLEMENTARY INFORMATION FILE</u> to:**

# PAXX and its paralogues synergistically direct DNA polymerase λ activity in DNA repair.

Andrew Craxton[1], Deeksha Munnur[1,2], Rebekah Jukes-Jones[1], George Skalka[1], Claudia Langlais[1], Kelvin Cain[1] and Michal Malewicz[1,*]

[1] MRC Toxicology Unit, Hodgkin Building, Lancaster Road, Leicester, LE1 9HN, UK.

[2] Current address: Sir William Dunn School of Pathology, South Parks Road, University of Oxford, Oxford, UK

Running title: PAXX and its paralogs regulate DNA Polymerase λ function in NHEJ

[*]To whom correspondence should be addressed:

Michal Malewicz, MRC Toxicology Unit, Hodgkin Building, Lancaster Road, LE1 9HN Leicester, UK; tel: 00441162525579; fax: 00441162525616; e-mail: mzm23@mrc-tox.cam.ac.uk

# Supplementary Information File

## Supplementary Figure 1

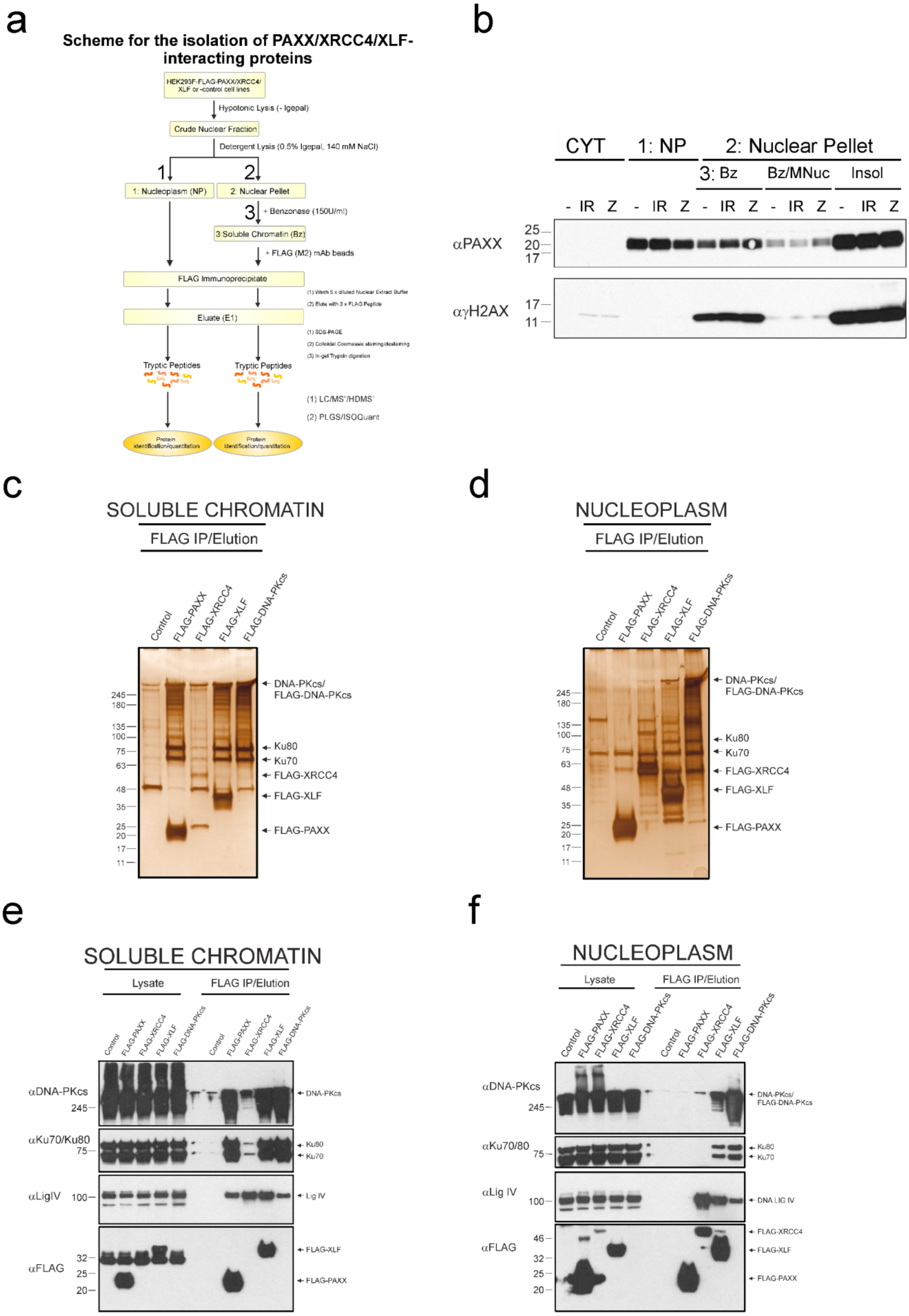

**Supplementary Figure 1: Comparative Analysis of the Proteomes of XRCC4 Family Proteins.** (a): Schematic figure showing a flow chart for the identification of FLAG-tagged PAXX, -XLF and –XRCC4 associated proteins by affinity chromatography and mass spectrometry from benzonase-treated "soluble chromatin" and nucleoplasmic fractions from HEK293 cells. (b): Untreated (-), irradiated (IR, 10Gy) or Zeocin (Z)-treated HEK293F cells were fractionated into non-nuclear soluble (CYT) or nuclear soluble (NP) or nuclear pellets. Nuclear pellets were either incubated with benzonase (Bz) or a combination of benzonase and micrococcal nuclease (Bz/MNuc). Fractions were resolved by SDS-PAGE and immunoblotted with the indicated antibodies. (c-d): Eluates of purified anti-FLAG IPs from (c) soluble chromatin or (d) nucleoplasmic fractions isolated from HEK293F-control, -FLAG-PAXX, -XLF, -XRCC4 or DNA-PKcs-expressing cells were resolved by SDS-PAGE and analysed by silver-staining. (e-f): As above, except the indicated NHEJ proteins were detected by immunoblotting using (e) soluble chromatin or (f) nucleoplasmic fractions.

**Supplementary Figure 2**

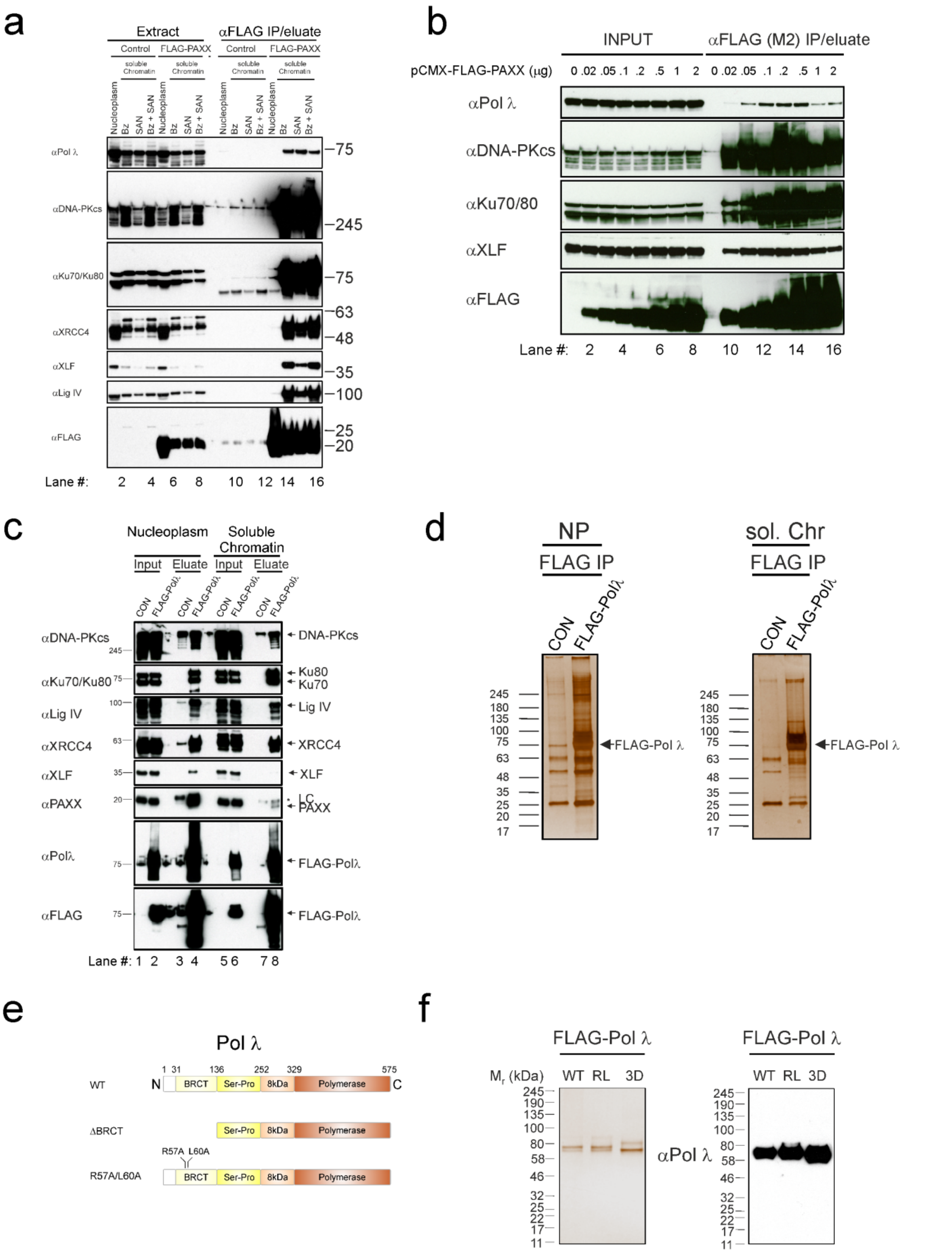


**Supplementary Figure 2: Pol λ interacts with endogenous PAXX and its paralogs in cells.**

(a): HEK293F or (b): U2OS cells were transiently transfected with either pCMX-LacZ (Control) or pCMX-FLAG-PAXX and nucleoplasmic or soluble extracts from benzonase- and/or salt active

nuclease (SAN)-treated nuclear pellets were isolated. Following anti-FLAG IP and FLAG peptide elution, FLAG-PAXX and associated proteins were eluted, resolved by SDS-PAGE and immunoblotted for the indicated proteins. (c): As described in Panel A, except that HEK293F cells were transiently transfected with either pCMX-LacZ (Control) or pCMX-FLAG-Pol λ. (d): Extracts from either nucleoplasmic (NP) or soluble chromatin (sol. Chr.) fractions or eluates of purified anti-FLAG IPs from HEK293F cells transiently transfected with either empty vector or FLAG-Pol λ were resolved by SDS-PAGE and visualized by silver staining. The position of FLAG-Pol λ was confirmed by immunoblotting and is indicated by a black arrow. (e): Schematic representation of Pol λ BRCT domain deletion mutant and α–helix 1 R57A/L60A mutant. (f): Silver-stained gel and anti-FLAG immunoblot for purified human Pol λ-WT, -R57A/L60A and -D427A/D429A/D490A.

**Supplementary Figure 3**

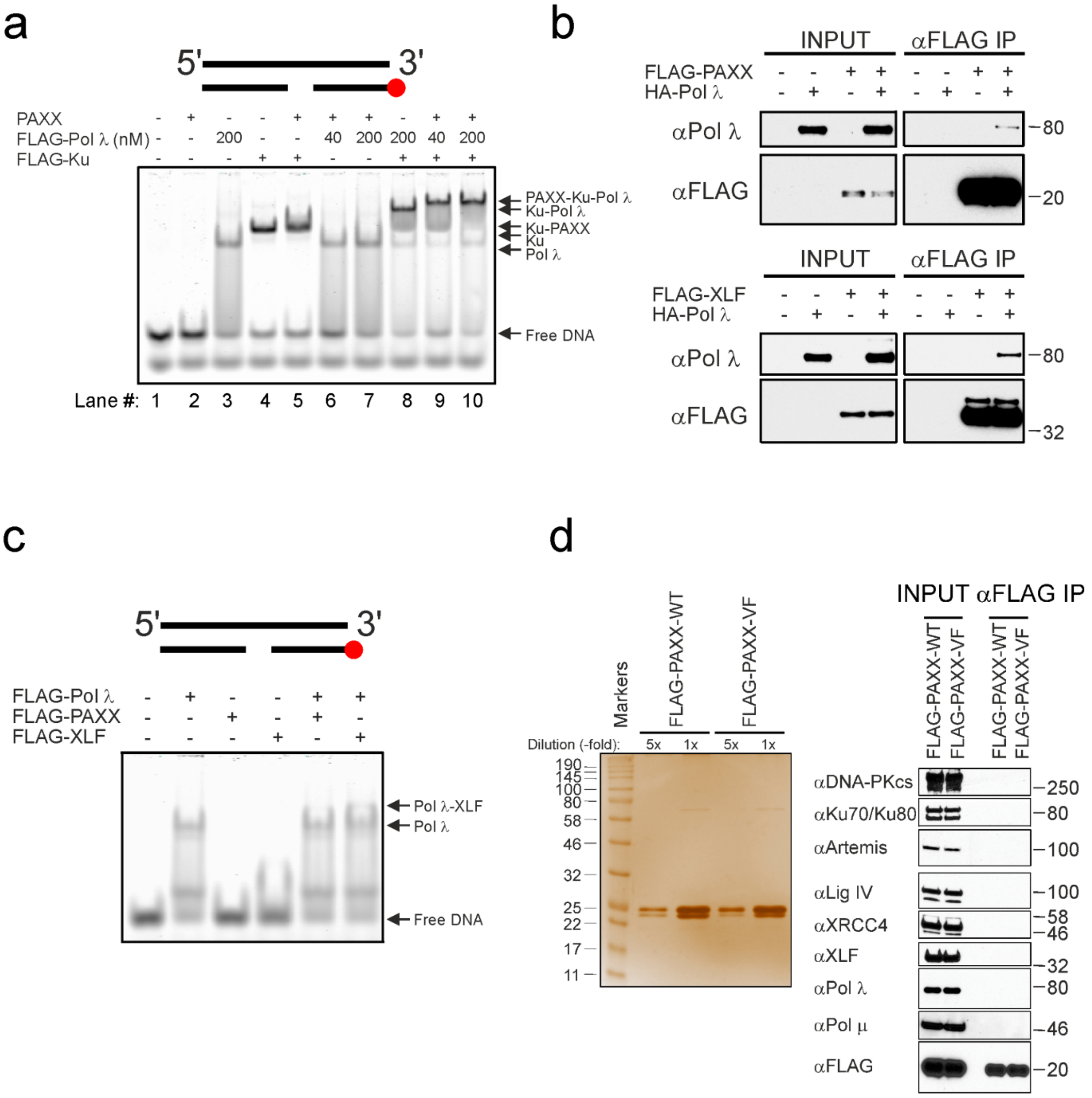


**Supplementary Figure 3: Interaction of PAXX with Pol λ requires the Ku binding motif of PAXX.** (a): EMSA showing that interaction of PAXX with Pol λ requires DNA-bound Ku. Reactions were performed with 20 nM IRDye® 700-labelled 5nt-gapped dsDNA (30-mer) and the following concentrations of FLAG-Ku70/80 (20 nM), FLAG-Pol λ (40 or 200 nM) or cleaved PAXX (0.5 µM). (b): *In vitro* interaction of PAXX or XLF with Pol λ. Affinity purified FLAG-PAXX (*upper panel)* or FLAG-XLF (*lower panel*) were mixed with affinity purified HA- Pol λ and immunoprecipitated with FLAG (M2)-agarose. Following FLAG peptide elution, eluates were immunoblotted with the

indicated antibodies. (c): As shown above in Panel A, except that reactions contained the indicated proteins (d): Silver-stained gel (*left* panel) and immunoblots (*right* panel) of purified FLAG-tagged human PAXX-WT or a V199A/F201A (VF) mutant.  Immunoblots were probed with the indicated antibodies.

**Supplementary Figure 4**

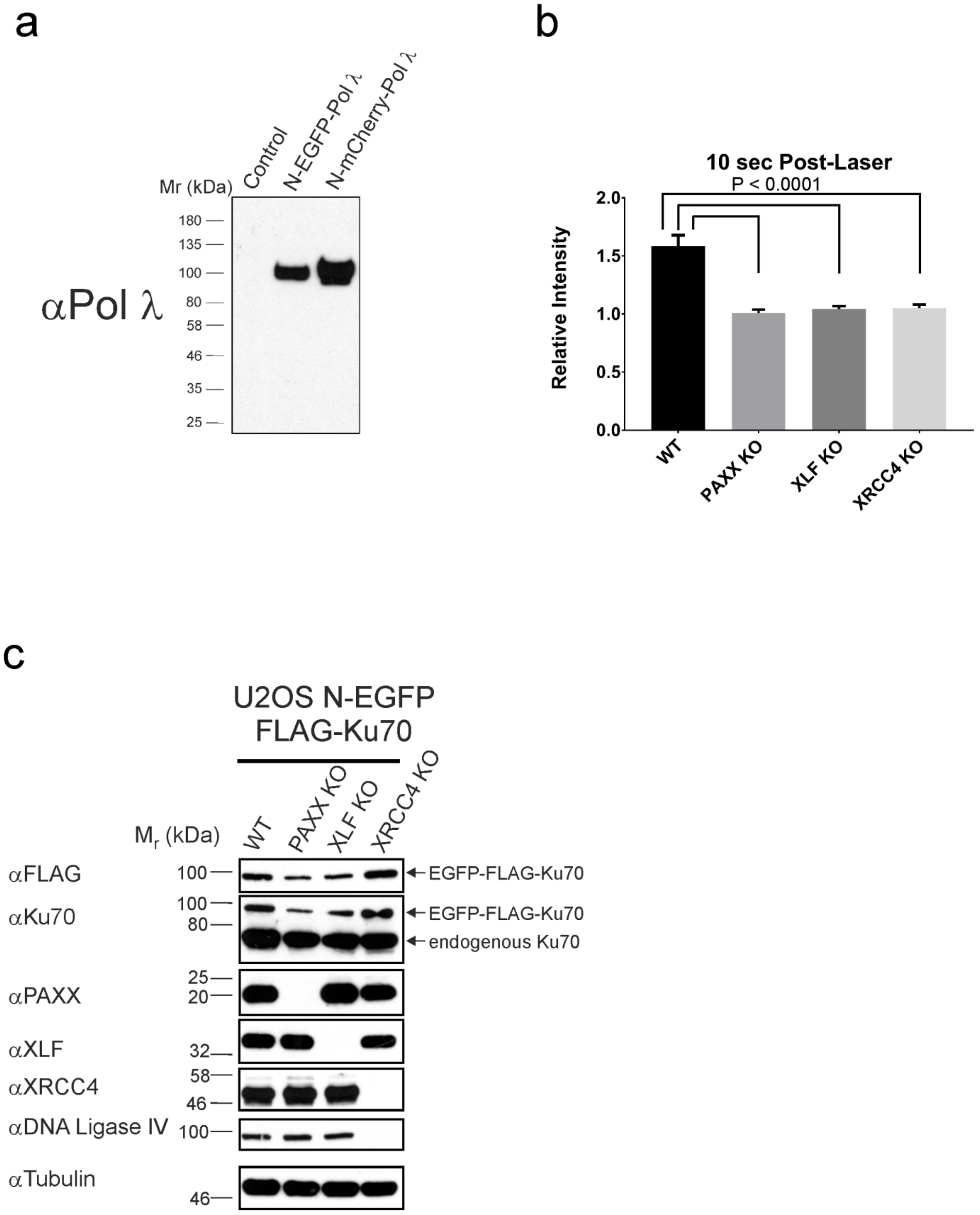


**Supplementary Figure 4: Role of PAXX and its paralogs in the recruitment of Pol λ to laser microirradiation-induced DNA damage sites.** (a): Immunoblot showing N-EGFP- and -mCherry-Pol λ fusion proteins are expressed as 100kDa proteins in U2OS cells. (b): Bar graph indicates the

mean ± SEM for normalised N-EGFP- Pol λ fluorescence at the laser-induced microirradiation site 10 sec following laser pulse. P-values were calculated using a 2-tailed students t-test with Graphpad. (c): Immunoblot analysis of N-EGFP-FLAG-Ku70-expressing U2OS cells deficient in PAXX, XLF or XRCC4 generated by CRISPR-Cas9. WCL were resolved by SDS-PAGE and the indicated proteins detected by immunoblotting.

**Supplementary Figure 5**

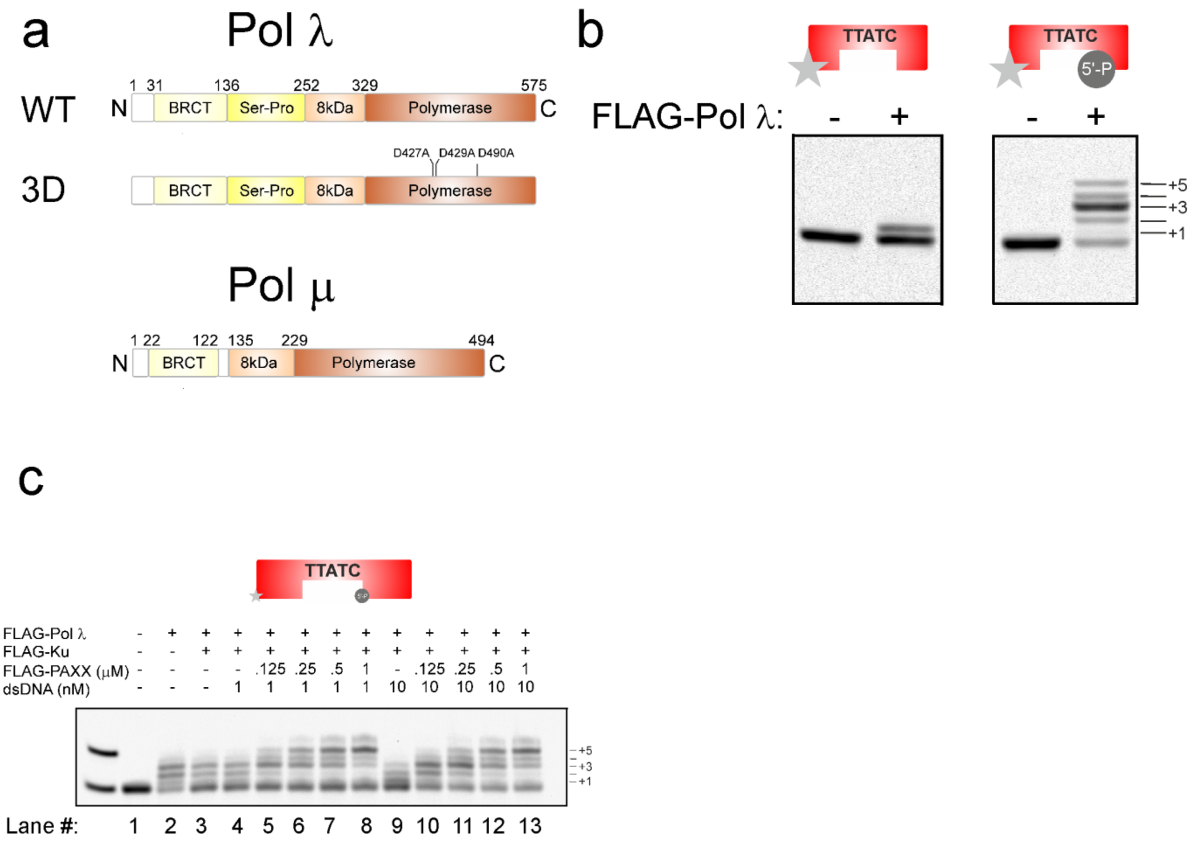


**Supplementary Figure 5: PAXX stimulates Pol λ-dependent gap filling synthesis activity in the presence of Ku** (a): Schematic representation of catalytically-inactive Pol λ D427A/D429A/D490A mutant and Pol μ. (b): Gap-filling synthesis activity of FLAG-Pol λ-WT using either an IRDye® 700-labelled non-phosphorylated (*left* panel) or 5'-phosphorylated (*right* panel) 5nt-gapped dsDNA (33-mer) substrate. (c): PAXX enhances Pol λ-dependent gap filling synthesis activity in a concentration-dependent manner in the presence of Ku.

**Supplementary Figure 6**

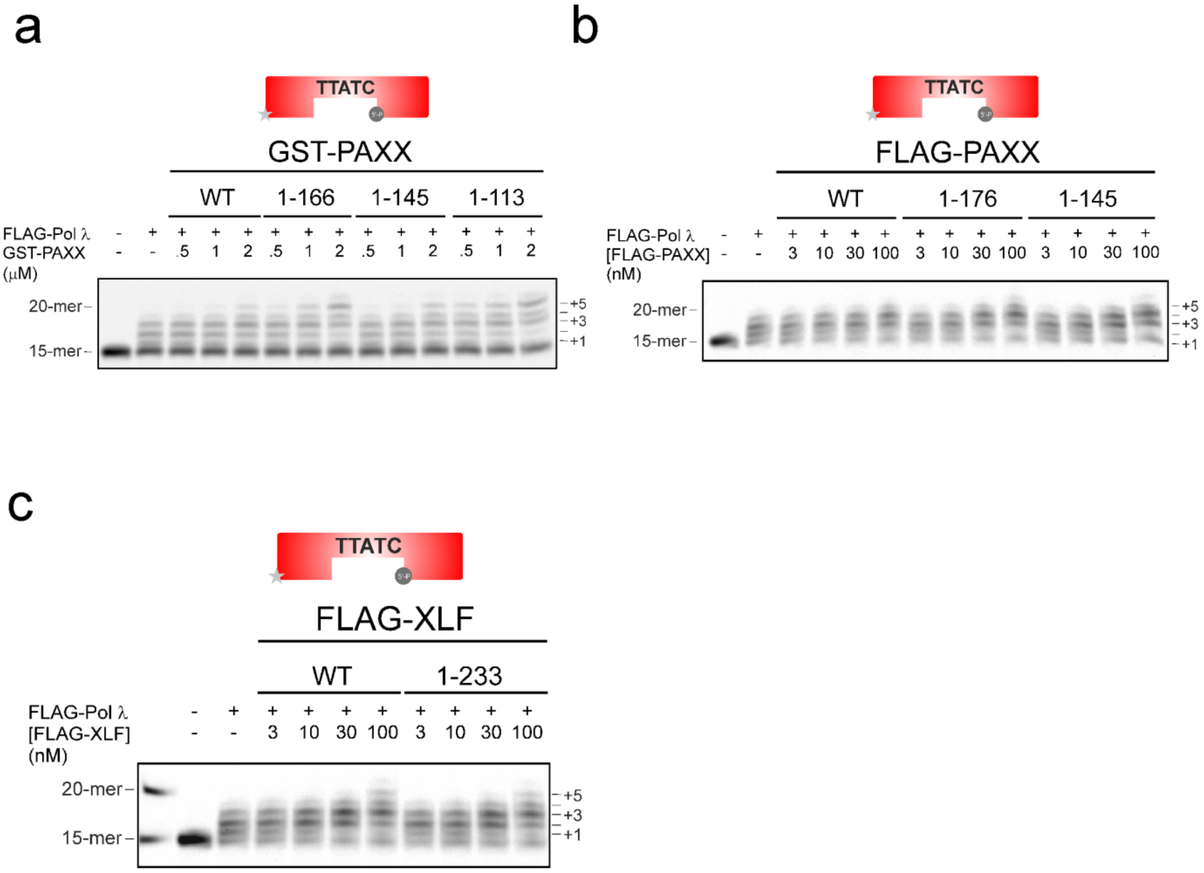


**Supplementary Figure 6: C-terminal regions of PAXX and XLF are not required for stimulation of Pol λ-dependent gap filling synthesis activity.** (a): Effect of GST-PAXX C-terminal deletion mutants on Pol λ-dependent gap filling synthesis activity. (b): Stimulation of Pol λ-dependent gap filling synthesis activity by PAXX is not affected by deletion of its C-terminal 59 amino acids. (c): Stimulation of Pol λ-dependent gap filling synthesis activity by XLF is not affected by deletion of its C-terminal 66 amino acids.

**Supplementary Figure 7**

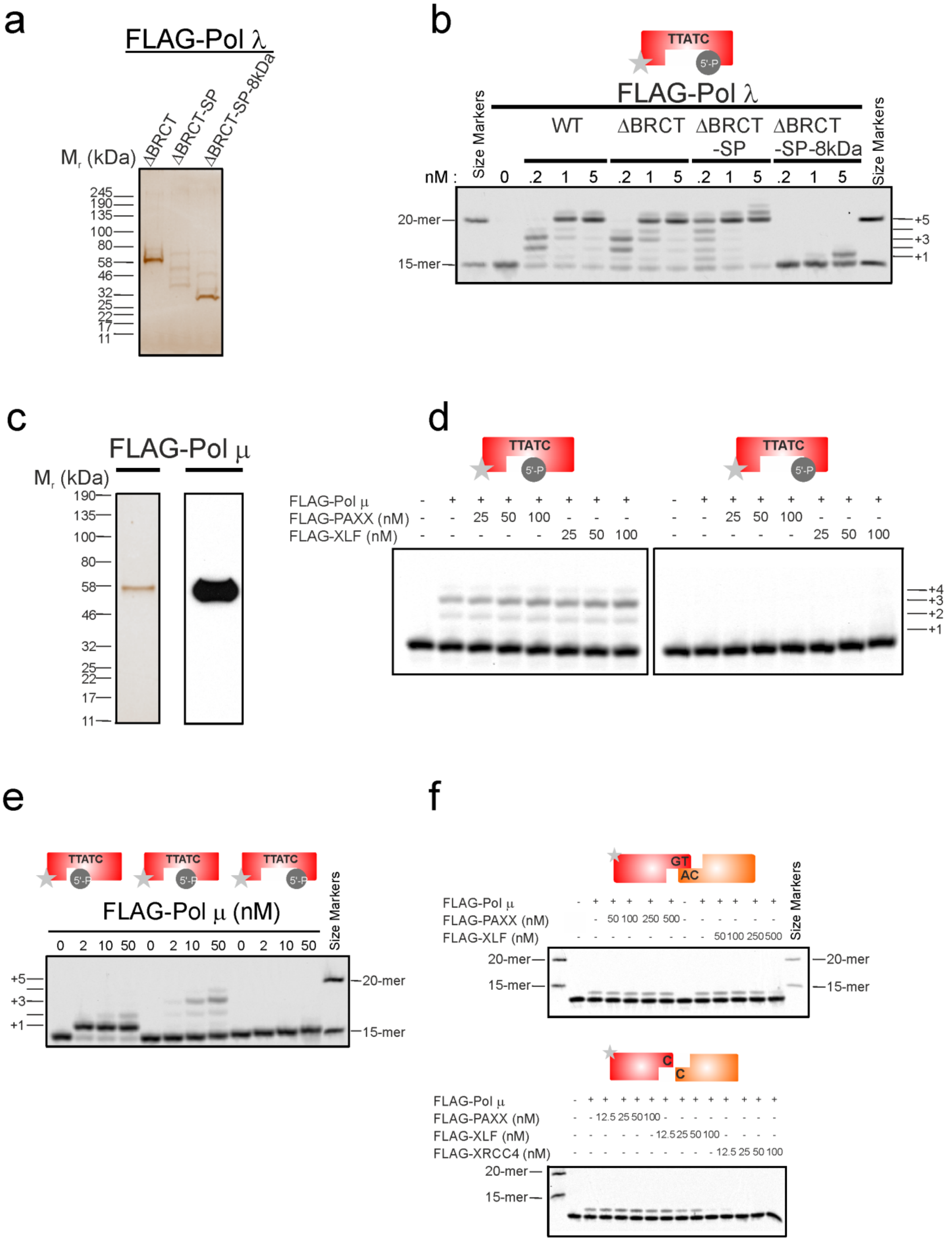


**Supplementary Figure 7: XRCC4 family proteins do not stimulate template-dependent or –independent activity of Pol μ.** (a): Silver-stained gel showing purified FLAG-tagged human Pol λ-

ΔBRCT, -ΔBRCT-Ser/Pro and ΔBRCT-Ser/Pro-8kDa domain mutants. (b): Gap-filling synthesis reactions were performed using an IRDye® 700-labelled 5'-phosphorylated 5nt-gapped dsDNA (33-mer) substrate with the indicated concentrations of FLAG-Pol λ-WT, -ΔBRCT, -ΔBRCT-Ser/Pro or ΔBRCT-Ser/Pro-8kDa domain mutants. (c): Silver-stained gel and FLAG immunoblot for purified human Pol μ. (d): Gap-filling synthesis reactions were performed with FLAG-tagged human Pol μ and IRDye® 700-labelled 3- or 5nt-gapped dsDNA (33-mer) substrates (*left* and *right* panels, respectively) with the indicated concentrations of FLAG-PAXX or –XLF (e): Gap-filling synthesis reactions were performed with IRDye® 700-labelled 1-, 3- or 5nt-gapped dsDNA (33-mer) substrates and FLAG-tagged human Pol μ (0-50 nM). IRDye®-800-labelled 15- and 20 nt oligonucleotides size markers are shown in the far-right lane. (f): Assays were performed using the indicated IRDye® 700-labelled NHEJ substrates containing either 1bp complementary template (*upper* panel) or no complementary template sequence (*lower* panel) with FLAG-Pol μ and the indicated concentrations of FLAG-PAXX, -XLF or –XRCC4.

**Supplementary Figure 8**

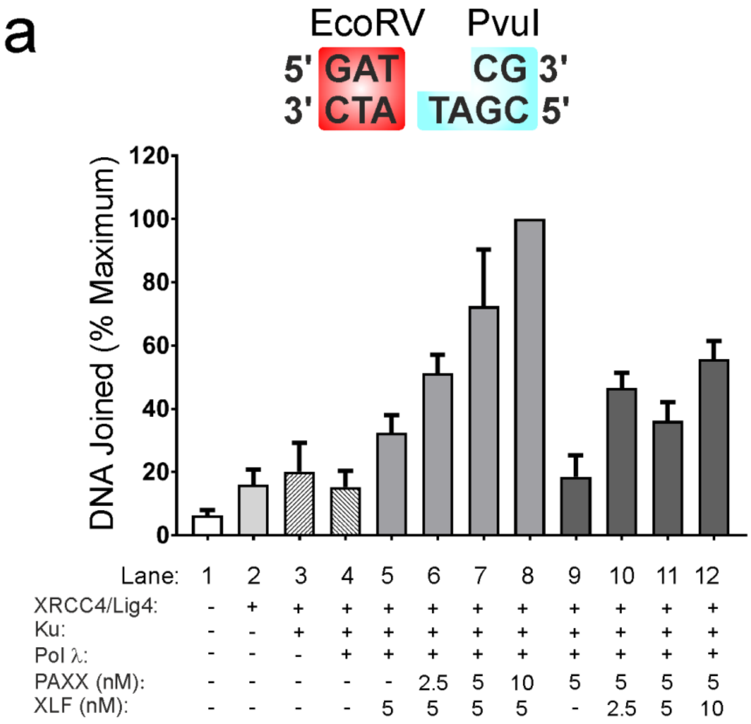


**Supplementary Fig. 8:** (a) PAXX and XLF synergise with Pol λ in a concentration-dependent manner to promote ligation of non-cohesive DNA ends which require Pol λ gap filling activity. Linear EcoRV-PvuI blunt-2nt 3' overhang DNA substrate was incubated with the indicated combinations of XRCC4/Lig IV, Ku70/80, PAXX, XLF and Pol λ and the joining efficiency quantified by qPCR with a TaqMan probe using a standard curve of $\log_{10}$ % joining efficiency versus $C_t$ value generated using prejoined DNA fragments. Results shown are the mean ± SEM from 3 experiments performed in triplicate. Samples in lanes 7 and 11 contain the same reaction components, except for the order of addition of PAXX and XLF.

**Supplementary Figure 9**

Supplementary Figure 9

Fig. 3a

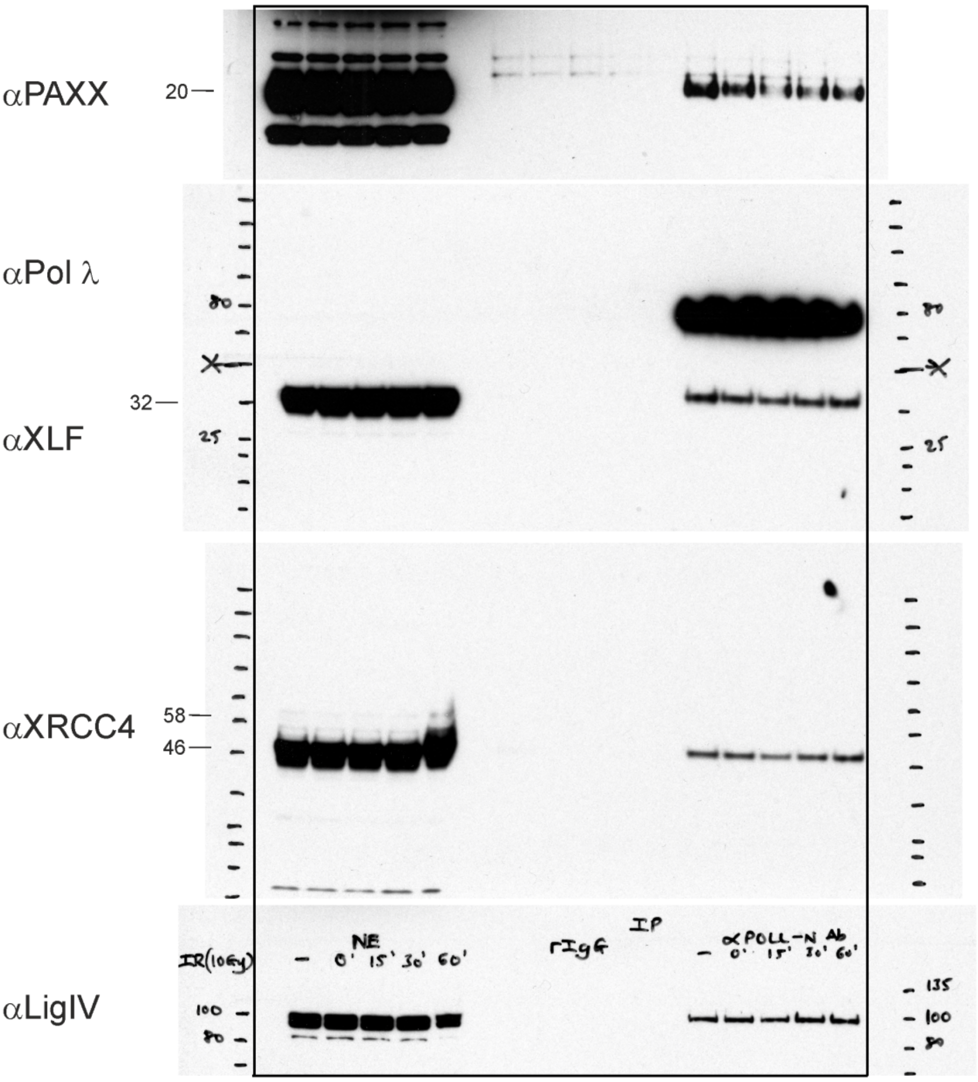

# Supplementary Figure 9

Fig. 3b

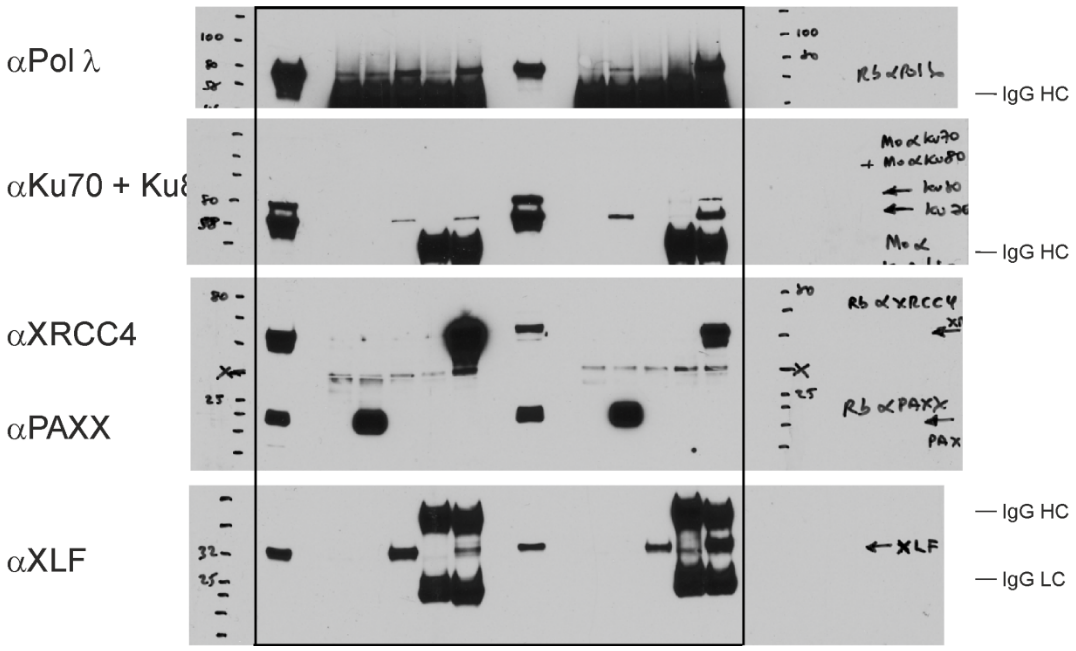

# Supplementary Figure 9

## Fig. 3c

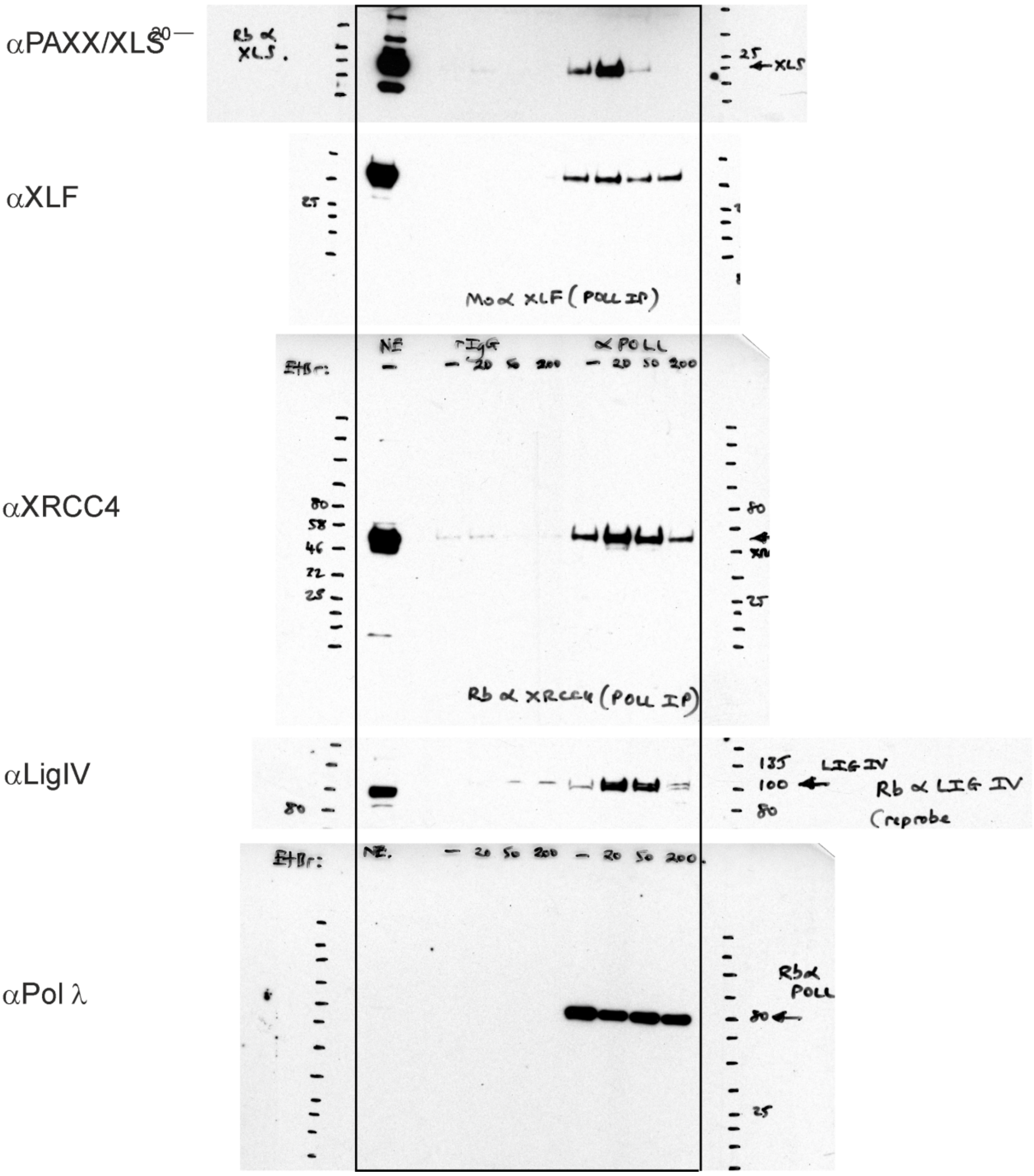

## Supplementary Figure 9

Fig. 3e

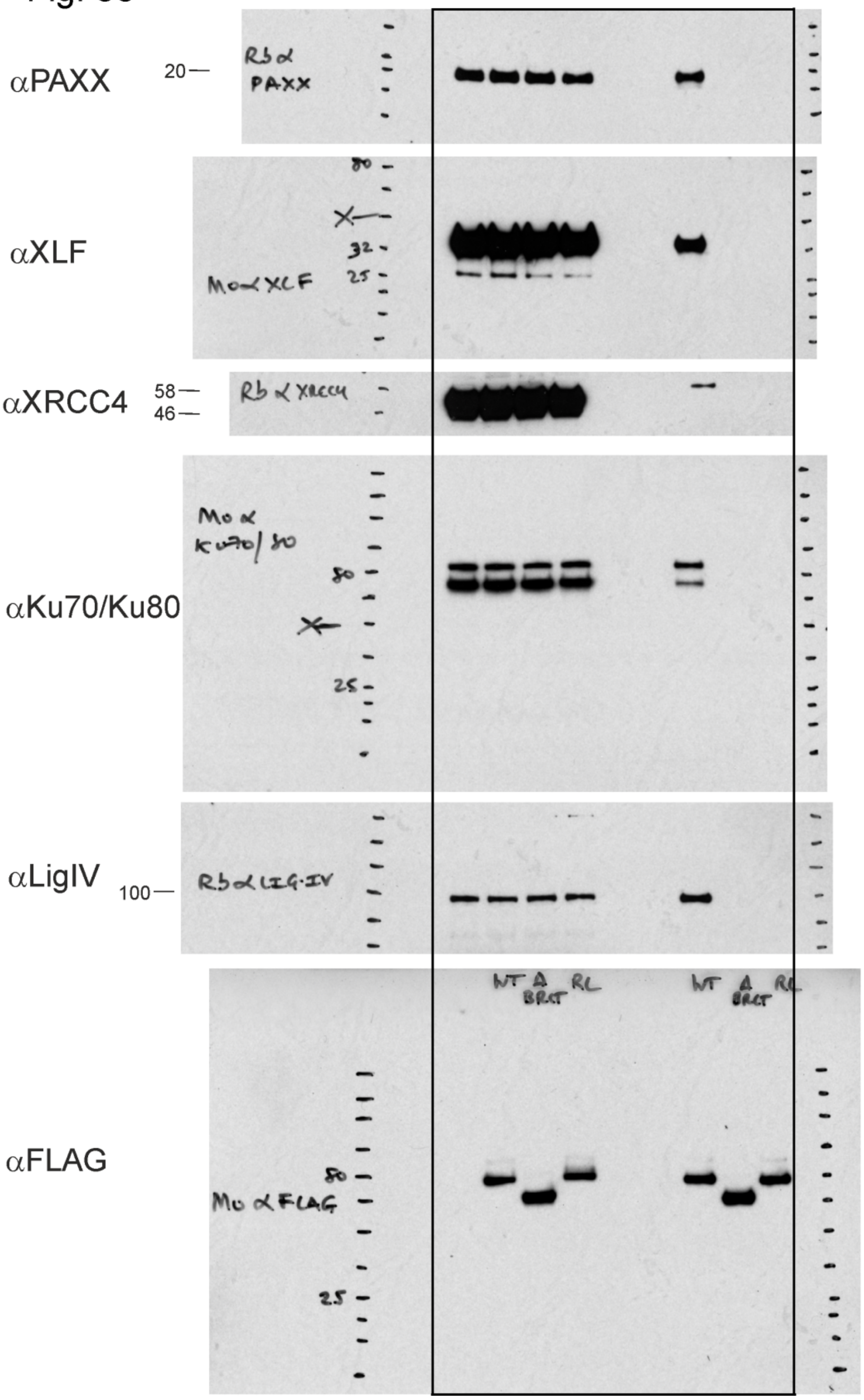

# Supplementary Figure 9

Fig. 5d

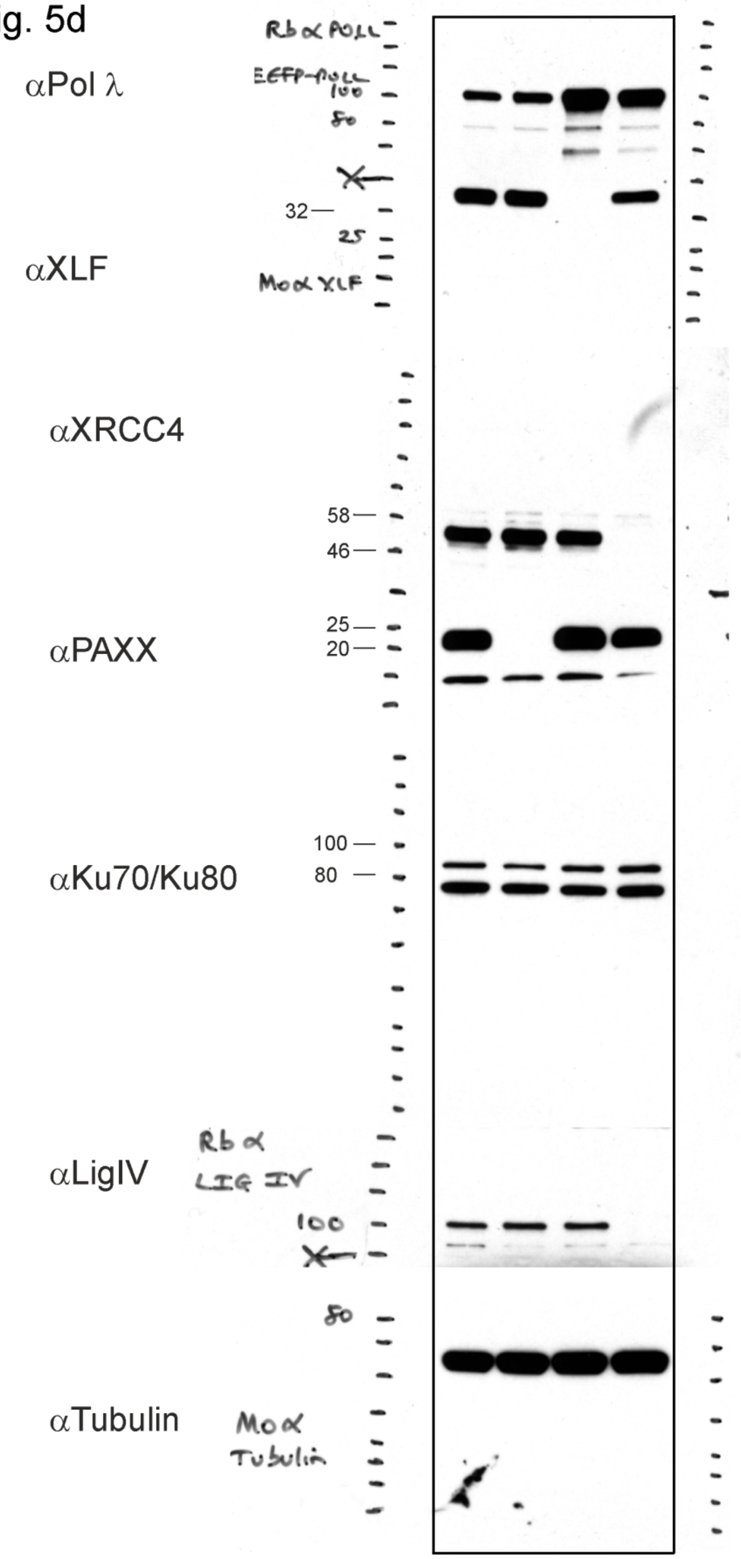

## Supplementary Figure 9

Fig. 7c-left panel

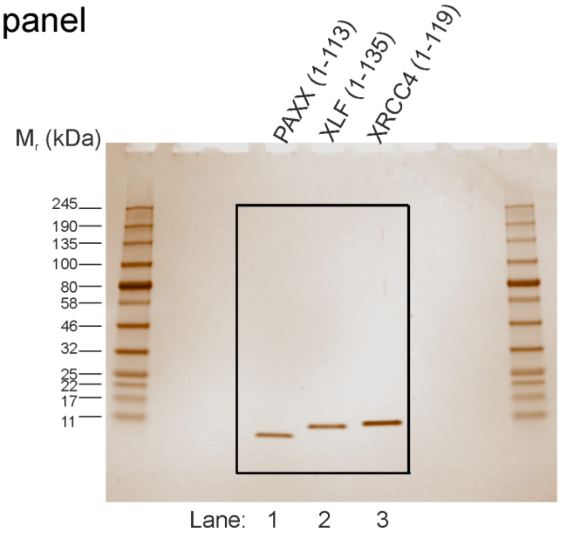


Fig. 7c-right panel

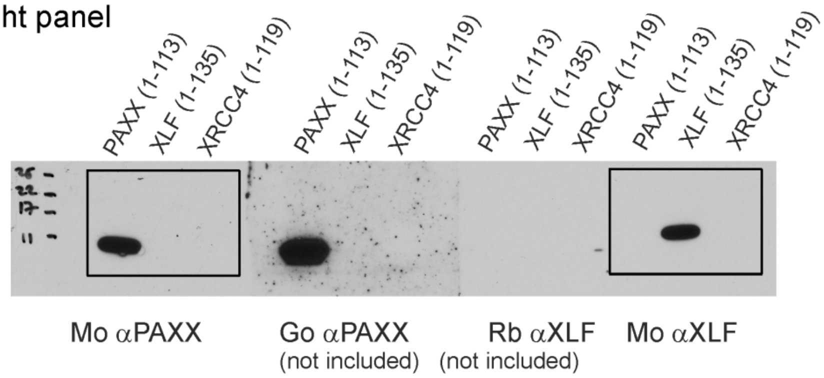


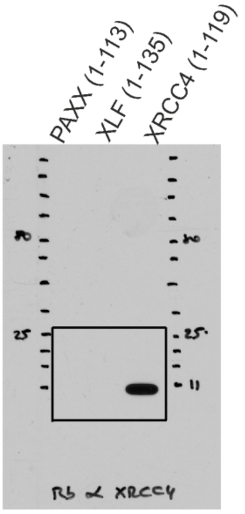

Supplementary Figure 9

Fig. 10a

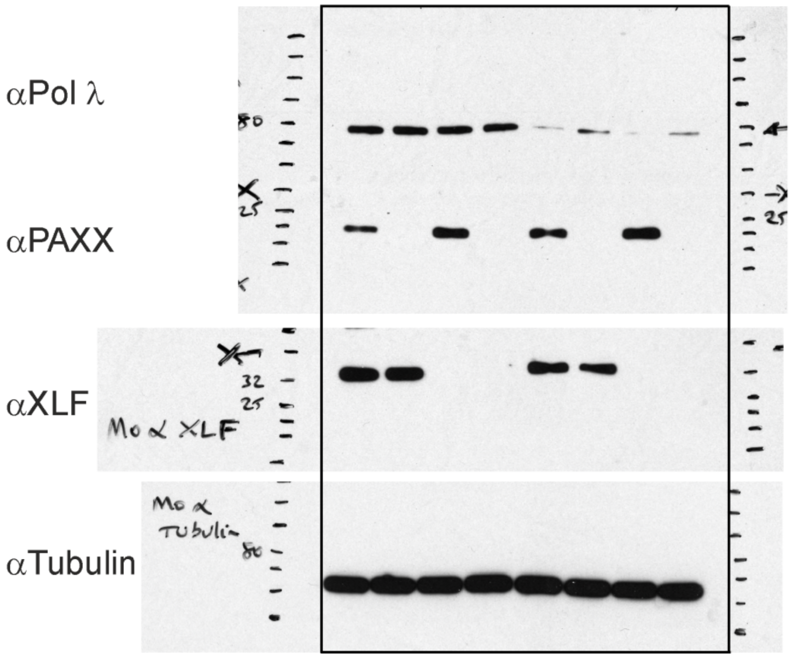

**Supplementary Table 1:**

| DNA Substrate | Primer | Sequence |
|---|---|---|
| **5nt gap 33-mer**<br>(5'-phosphorylated) | 33-mer | 5'-ACTGGCCGTCGTTCTATTGTACTCACTGTGATC-3' |
| | 15-mer | 5'- IRDye® 700 -GATCACAGTGAGTAC-3' |
| | 13-mer | 5'-pAACGACGGCCAGT-3' |
| **3nt gap 33-mer**<br>(5'-phosphorylated) | 33-mer | As above |
| | 15-mer | As above |
| | 15-mer | 5'-pAGAACGACGGCCAGT-3' |
| **1nt gap 33-mer** | 33-mer | As above |
| (5'-phosphorylated) | 15-mer | As above |
| | 17-mer | 5'-pATAGAACGACGGCCAGT-3' |
| **NHEJ**<br>(template-dependent) | 13-mer | 5'- IRDye® 700 –CCCTCCCTCCCGT-3' |
| | 11-mer | 5'-GGGAGGGAGGG-3' |
| | 15-mer | 5'-GCACTCACGTCCCCA-3' |
| | 13-mer | 5'-GGGACGTGAGTGC-3' |
| **NHEJ**<br>(template-independent) | 12-mer | 5'- IRDye® 700 –CCCTCCCTCCCC-3' |
| | 11-mer | 5'-GGGAGGGAGGG-3' |
| | 14-mer | 5'-GCACTCACGTCCCC-3' |
| | 13-mer | 5'-GGGACGTGAGTGC-3' |

**Supplementary Table 2:**

| Primer | FWD/REV | Sequence |
|---|---|---|
| **EcoRI-DNA1** | **FWD** | CGGAATTCACAATTAATAGACTGGATGG |
| **EcoRI-DNA1** | **REV** | CGGAATTCATCTCAGTTCGGTGTAGGTC |
| **KpnI-DNA1** | **FWD** | CGGGTACCACAATTAATAGACTGGATGG |
| **KpnI-DNA1** | **REV** | CGGGTACCATCTCAGTTCGGTGTAGGTC |
| **EcoRV-DNA1** | **FWD** | CGGATATCACAATTAATAGACTGGATGG |
| **EcoRV-DNA1** | **REV** | CGGATATCATCTCAGTTCGGTGTAGGTC |
| **BstEII-DNA1** | **FWD** | CGGGTGACCACAATTAATAGACTGGATGG |
| **BstEII-DNA1** | **REV** | CGGGTGACCATCTCAGTTCGGTGTAGGTC |
| **PvuII-DNA1** | **FWD** | CGCGATCGACAATTAATAGACTGGATGG |
| **PvuII-DNA1** | **REV** | CGCGATCGATCTCAGTTCGGTGTAGGTC |

**Supplementary Table 3:**

| Primer | FWD/REV | Sequence |
|---|---|---|
| **EcoRI-DNA2** | **FWD** | CGGAATTCGGAGCTTCCAGGGGGAAACG |
| **EcoRI-DNA2** | **REV** | CGGAATTCGGCAGCTTCCACAGCAATGG |
| **KpnI-DNA2** | **FWD** | CGGGTACCGGAGCTTCCAGGGGGAAACG |
| **KpnI-DNA2** | **REV** | CGGGTACCGGCAGCTTCCACAGCAATGG |
| **EcoRV-DNA2** | **FWD** | CGGATATCGGAGCTTCCAGGGGGAAACG |
| **EcoRV-DNA2** | **REV** | CGGATATCGGCAGCTTCCACAGCAATGG |
| **BstEII-DNA2** | **FWD** | CGGGTGACCGGAGCTTCCAGGGGGAAACG |
| **BstEII-DNA2** | **REV** | CGGGTGACCGGCAGCTTCCACAGCAATGG |
| **PvuII-DNA2** | **FWD** | CGCGATCGGGAGCTTCCAGGGGGAAACG |
| **PvuII-DNA2** | **REV** | CGCGATCGGGCAGCTTCCACAGCAATGG |

**Supplementary Table 4:**

| Gene | Exon | Target Sequence |
|---|---|---|
| **PAXX** | 1 | GTGCAGAGCGGCGGCGACAG |
| **XLF/NHEJ1** | 7 | TTGGCTATCGATTCCTTGCA |
| **XLF/NHEJ1** | 3 | TGCATCTGCCACACAATCAC |
| **XRCC4** | 4 | GTTCTCTAATGACTTCAGCT |

**Supplementary Table 5:**

| **Cell Line** | **Clone** | **Indel Size (nt)** | **Indel Sequence** |
|---|---|---|---|
| PAXX KO | 11 | del 37<br>del 35 | GGATCCGCTGTCGCCGCCGCTCTGCACGCTGCCGCCG<br><br>CCGCTGTCGCCGCCGCTCTGCACGCTGCCGCCGGG |
| XLF KO | 2-22 | del 2<br>del 163 | TT<br><br>TCCTTGCAGGGAAGCACTGTTTGAGGTATGAGGATCT<br>CCTGAAATCAGAAAGATCAAGAGAAGATAGTGATAA<br>ATGCCTTCTTCTTACCAGGAAGATATTAGCATTCACC<br>AAGCATTCTGATAAAGGCCAGGGTTTGGTACAAATAT<br>ATAAGCAGACAGAAGT |
| XRCC4 KO | 1-12 | in 1<br>del 3 (+ 4 nt change) | T<br><br>CCAGCTG to AAAT |
| PAXX/ XLF DKO | 2-1 | del 225<br><br>in 1 | CACAAATATCCCTCAATATTTTTGAGGCAGTGCCAATGAAGCAGC<br>AATTCCAGGGACCACTGGCTTGGGGGCTATAGGTGCCAACTCTCA<br>GAACACCATCCAGGAAGCTGCTCATGACTCACCGTGGACTCTTTC<br>TCAGGTGCTGAGAGGGTTGGGGCTGAGGAGACCAGTTGTTCTGG<br>CTGGTTTACACATTGGCTATCGATTCCTTGCAGGGAAGCACTGTT<br>T |

**Supplementary Table 6: NHEJ-related Proteins identified in purified FLAG IPs for FLAG-PAXX, -XRCC4, -XLF and –DNA-PKcs in the Benzonase-treated Nuclear Fraction identified by Mass Spectrometry**

| | **Bait Protein** | | | | |
|---|---|---|---|---|---|
| **Identified Protein (fmol)** | **Control** | **PAXX** | **XRCC4** | **XLF** | **DNA-PKcs** |
| **DNA-PKcs** | 46.8 | 1591.2 | 203.3 | 1454.2 | 1745 |
| **Ku70** | 31.7 | 1067.2 | 106 | 813.8 | 984 |
| **Ku80** | 39.7 | 825.7 | 150.8 | 792.5 | 1026.9 |
| **Lig IV** | 0 | 56.3 | 67 | 203.9 | 10 |
| **XRCC4** | 4.8 | 73.1 | 412 | 203.4 | 55 |
| **XLF** | 0 | 74.6 | 64.9 | 777.3 | 122.2 |
| **PAXX** | 112 | 1026.3 | 118.5 | 162.9 | 156.6 |
| **PARP1** | 34.4 | 81.5 | 8 | 51 | 63.9 |
| **PNKP** | 0 | 0 | 3.5 | 15.7 | 0 |
| **WRN** | 0 | 81.6 | 0 | 47.7 | 129 |
| **Pol λ** | 0 | 53.2 | 4.1 | 38.9 | 0 |
| **Artemis** | 0 | 0 | 0 | 0 | 0 |
| **APTX** | 0 | 0 | 7.5 | 0 | 0 |
| **APLF** | 0 | 0 | 0 | 6.8 | 0 |

Results shown are the averages (fmol) from duplicate technical repeats of two separate experiments. FLAG-tagged bait NHEJ factors and Pol λ are highlighted by shaded yellow and pale yellow boxes respectively. Abbreviations used are as follows: DNA-PKcs, DNA-dependent protein kinase catalytic subunit; Lig IV, DNA ligase IV; XLF, XRCC4-like factor; PAXX, paralog of XRCC4 and XLF; PARP1, poly(ADP-ribose) polymerase 1; PNKP, bifunctional polynucleotide phosphatase/kinase; WRN, Werner helicase; Pol λ, DNA polymerase lambda; APTX, Aprataxin; APLF, Aprataxin- and PNK-like factor.

**Supplementary Table 7: NHEJ-related Proteins identified in purified FLAG IPs for FLAG-PAXX, -XRCC4, -XLF and –DNA-PKcs in the Nucleoplasmic Fraction identified by Mass Spectrometry**

| | **Bait Protein** | | | | |
|---|---|---|---|---|---|
| **Identified Protein (fmol)** | **Control** | **PAXX** | **XRCC4** | **XLF** | **DNA-PKcs** |
| **DNA-PKcs** | 0 | 0 | 20.1 | 254.3 | 1451 |
| **Ku70** | 2.8 | 10.1 | 22 | 180.6 | 396 |
| **Ku80** | 0 | 10.9 | 19 | 147.9 | 277.6 |
| **Lig IV** | 0 | 0 | 139.1 | 188.9 | 158.9 |
| **XRCC4** | 17.3 | 37.2 | 1396 | 554.2 | 89.9 |
| **XLF** | 0 | 9.1 | 133.6 | 1354.6 | 94.8 |
| **PAXX** | 143.7 | 1379 | 79.3 | 110.2 | 130.2 |
| **PARP1** | 0 | 0 | 12.2 | 101 | 145.9 |
| **PNKP** | 0 | 0 | 65.8 | 39.8 | 11.4 |
| **WRN** | 0 | 0 | 0 | 0 | 63.3 |
| **Pol λ** | 0 | 0 | 0 | 5.3 | 0 |
| **Artemis** | 0 | 0 | 0 | 0 | 20.3 |
| **APTX** | 0 | 0 | 41.7 | 0 | 0 |
| **APLF** | 0 | 0 | 0 | 0 | 0 |

Results shown are the averages (fmol) from duplicate technical repeats of two separate experiments. FLAG-tagged bait NHEJ factors and Pol λ are highlighted by shaded yellow and pale yellow boxes respectively.

**Supplementary Table 8: Proteins identified by Mass Spectrometry which selectively interact with PAXX or XLF[1, 2]**

| PAXX | | XLF | |
|---|---|---|---|
| **Nucleoplasm** | **Soluble Chromatin** | **Nucleoplasm** | **Soluble Chromatin** |
| | KV201_HUMAN | SP16H_HUMAN | KV206_HUMAN |
| | DYHC2_HUMAN | SSRP1_HUMAN | H2A2B_HUMAN |
| | MYOME_HUMAN | LAP2A_HUMAN | APOB_HUMAN |
| | PARK7_HUMAN | PNKP_HUMAN | PNKP_HUMAN |
| | PP6R3_HUMAN | KPYM_HUMAN | |
| | SMEK3_HUMAN | OGFD1_HUMAN | |
| | NEBU_HUMAN | RFA1_HUMAN | |
| | CREB1_HUMAN | SEP14_HUMAN | |
| | RYR3_HUMAN | IFFO1_HUMAN | |
| | CK5P2_HUMAN | PDIA1_HUMAN | |
| | | EMD_HUMAN | |
| | | IFFO2_HUMAN | |
| | | BAF_HUMAN | |
| | | TCPB_HUMAN | |
| | | P4HA1_HUMAN | |
| | | | |
| | | | |
| | | | |

[1]Proteins listed include only those detected above an average of > 10 fmol in 2 experiments (duplicate technical repeats per expt).

[2]All keratin, tubulin and ribosomal species of proteins were excluded from the list.

**Supplementary Table 9: NHEJ-related Proteins identified in purified FLAG-Pol λ IPs of Nucleoplasmic and Benzonase-treated Fractions identified by Mass Spectrometry**

| | Nucleoplasm | | Benzonase-treated Soluble Chromatin | |
|---|---|---|---|---|
| **Identified Protein (fmol)** | **Control** | **Pol λ** | **Control** | **Pol λ** |
| **Pol λ** | 81.2 | 1400 | 96.7 | 557 |
| **DNA-PKcs** | 7.9 | 381.3 | 0 | 35.0 |
| **Ku70** | 19.2 | 371.0 | 0 | 206.1 |
| **Ku80** | 32.8 | 452.6 | 14 | 217.7 |
| **XRCC4** | 0 | 25.8 | 0 | 17.5 |
| **XLF** | 0 | 25.1 | 0 | 0 |
| **PAXX** | 0 | 47.9 | 0 | 0 |
| **Lig IV** | 0 | 21.5 | 0 | 36.3 |
| **PARP1** | 9.8 | 90.9 | 14.1 | 113.4 |
| **PNKP** | 0 | 0 | 0 | 119.0 |
| **WRN** | 0 | 13.6 | 0 | 0 |
| **Artemis** | 0 | 0 | 0 | 0 |
| **APTX** | 0 | 0 | 0 | 0 |
| **APLF** | 0 | 0 | 0 | 0 |

Results shown are the averages (fmol) from either duplicate or triplicate technical repeats of two separate experiments. FLAG-tagged Pol λ bait protein and XRCC4 paralogs are highlighted by shaded yellow and pale yellow boxes respectively. Abbreviations used are as follows: DNA-PKcs, DNA-dependent protein kinase catalytic subunit; Lig IV, DNA ligase IV; XLF, XRCC4-like factor; PAXX, paralog of XRCC4 and XLF; PARP1, poly(ADP-ribose) polymerase 1; PNKP, bifunctional polynucleotide phosphatase/kinase; WRN, Werner helicase; Pol λ, DNA polymerase lambda; APTX, Aprataxin; APLF, Aprataxin- and PNK-like factor.
