# Supplementary material for: PAXX and its paralogs synergistically direct DNA polymerase λ activity in DNA repair
Source: Nat Commun. 2018 Sep 24;9:3877. doi: 10.1038/s41467-018-06127-y (PMC6155126; doi:10.1038/s41467-018-06127-y)
Supplement: Supplementary file 3 — Description of Additional Supplementary Files [file 41467_2018_6127_MOESM3_ESM.pdf]

Description of Additional Supplementary Files

File Name: Supplementary Data 1
Description: Table of mass-spec proteomics data for PAXX, XLF, XRCC4 and DNA-PKcs proteins.

File Name: Supplementary Data 2
Description: Table of mass-spec proteomics data for polymerase lambda protein
